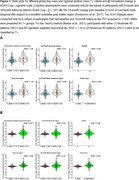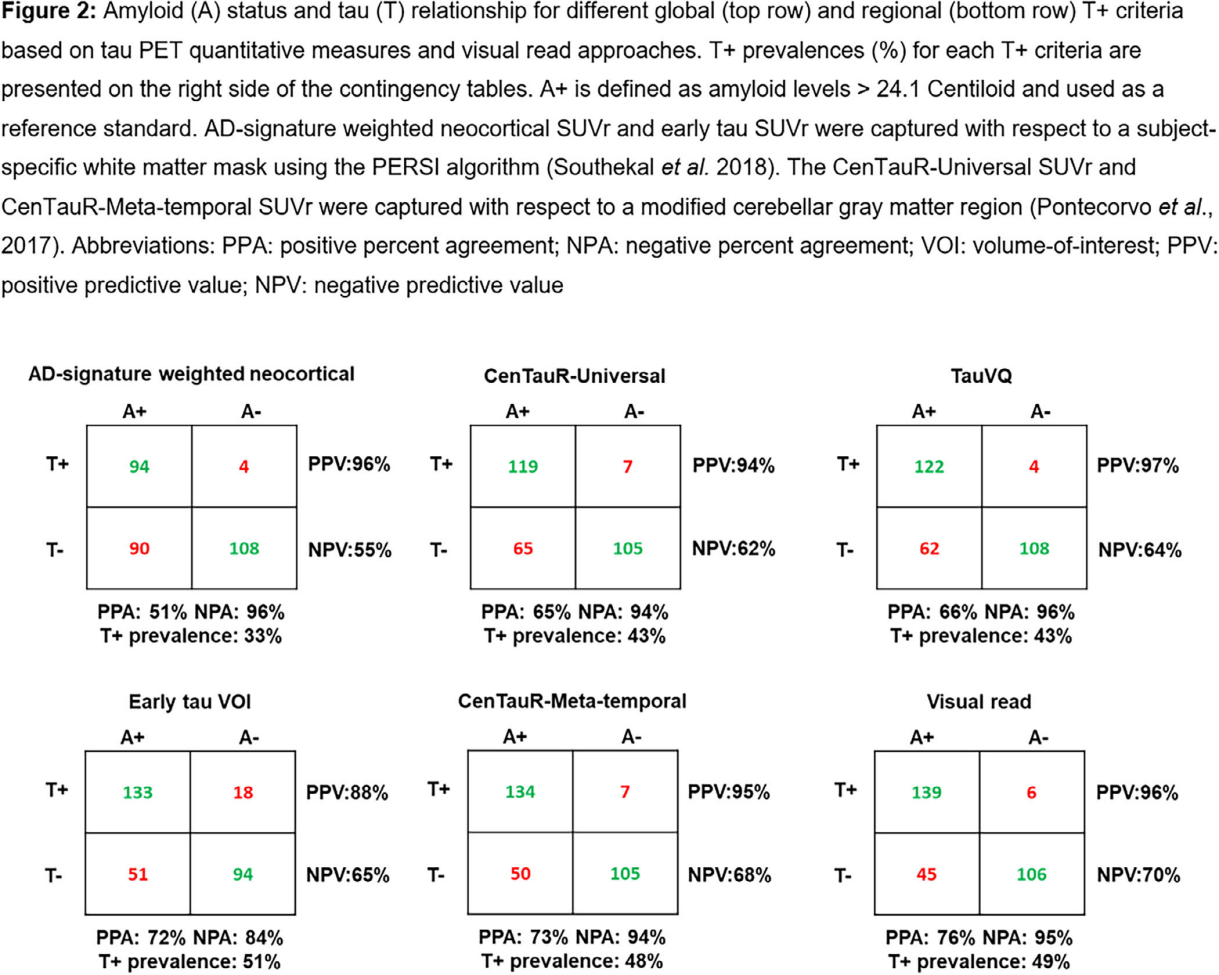# Quantitative‐ and visual read‐based flortaucipir PET T+ criteria for the pathophysiological assessment of Alzheimer’s Disease

**DOI:** 10.1002/alz.084516

**Published:** 2025-01-09

**Authors:** Ilke Tunali, Vikas Kotari, Ian A. Kennedy, Samantha C. Burnham, Sergey Shcherbinin

**Affiliations:** ^1^ Eli Lilly and Company, Indianapolis, IN USA

## Abstract

**Background:**

The revised NIA‐AA biological Alzheimer’s disease (AD) staging scheme considers various tau positivity (T+) rules based on global and regional neocortical tau PET uptakes. In AD clinical trials, various tau PET criteria (e.g., visual+quantitative [TauVQ], Mintun et al., 2021) have been employed to select eligible patients. In this study, we compared six T+ criteria in terms of their potential usefulness for biological staging and clinical trial eligibility.

**Methods:**

N=296 symptomatic (189/107 MCI/AD‐dementia) subjects from AV‐1451‐A05 (NCT02016560) were included. Six different tau‐PET stratifications (T+/T‐) were employed: Two quantitative global neocortical uptake measures (CenTauR‐Universal, Villemagne et al., 2023, and AD‐signature weighted neocortical, Devous et al., 2018); two quantitative temporal‐based uptake measures (CenTauR‐Meta‐temporal, Villemagne et al., 2023 and Early Tau VOI, Kotari et al., 2023), a visual read‐based method (Fleisher et al., 2021) and TauVQ method. A dataset of young cognitively normal subjects (N=16) was utilized to determine T+ thresholds for quantitative measures (mean+2.5xSD SUVr). The tau stratifications were evaluated for their agreement with amyloid‐PET positivity and for AD‐related prognostic utility using signal‐to‐noise ratios (SNRs) of T+ for (1) Annualized change in AD Assessment Scale‐Cognitive (ADAS‐Cog_11_) and (2) 18‐month change in CenTauR‐Meta‐temporal SUVr.

**Results:**

CenTauR‐Universal and CenTauR‐Meta‐Temporal derived T+ groups showed comparable SNRs (Figure 1A‐B) and agreement with amyloid positivity (Figure 2). The TauVQ‐ and the visual read‐derived T+ groups also performed similarly. The regional early tau VOI‐based T+ group, compared to the global AD‐signature neocortical‐based T+ group, showed a higher SNR for 18‐month change in CenTauR‐Meta‐temporal SUVr. However, a lower SNR for annualized ADAS‐Cog_11_ change and a lesser agreement with amyloid positivity were observed. Comparisons using PET‐to‐autopsy cohort will be presented.

**Conclusions:**

Our analyses suggest using CenTauR‐Meta‐temporal and visual read‐based regional T+ criteria may identify a larger subgroup of symptomatic patients exhibiting similar amyloid positivity prevalence and clinical decline compared to those determined by corresponding global T+ criteria. Among examined methods, the early tau VOI‐based T+ criterion possibly captures the earliest pathologic stage. Lastly, CenTauR‐based T+ groups showed lower SNRs for tau and clinical progression, suggesting flortaucipir‐specific criteria might be more sensitive for prognostic evaluations. Further validations using different datasets and cut points are warranted.